# Dengue virus serotype 4 infection in human fatal cases: histopathological investigation

**DOI:** 10.1590/0074-02760250248

**Published:** 2026-06-22

**Authors:** Arthur da Costa Rasinhas, Gabriela Cardoso Caldas, Ana Luisa Teixeira de Almeida, Fernanda Cunha Jácome, Marcos Alexandre Nunes da Silva, Milla Bezerra Paiva, Kíssila Rabelo, Jorge José de Carvalho, Felipe de Andrade Vieira Alves, Emília Tomé de Sousa, Ortrud Monika Barth, Flavia Barreto dos Santos

**Affiliations:** 1 Fundação Oswaldo Cruz-Fiocruz, Instituto Oswaldo Cruz, Laboratório das Interações Vírus-Hospedeiro, Rio de Janeiro, RJ, Brasil; 2 Fundação Oswaldo Cruz-Fiocruz, Instituto Oswaldo Cruz, Laboratório de Medicina Experimental e Saúde, Rio de Janeiro, RJ, Brasil; 3 Fundação Oswaldo Cruz-Fiocruz, Instituto Oswaldo Cruz, Laboratório de Morfologia e Morfogênese Viral, Rio de Janeiro, RJ, Brasil; 4 Universidade do Estado do Rio de Janeiro, Instituto de Biologia Roberto Alcântara Gomes, Laboratório de Ultraestrutura e Biologia de Tecidual, Rio de Janeiro, RJ, Brasil; 5 Fundação Oswaldo Cruz-Fiocruz, Instituto Oswaldo Cruz, Laboratório Interdisciplinar de Pesquisa Médica, Rio de Janeiro, RJ, Brasil; 6 Universidade Federal do Ceará, Departamento de Patologia e Medicina Forense, Fortaleza, CE, Brasil

**Keywords:** DENV-4, fatal cases, liver, lung, heart, kidney

## Abstract

**BACKGROUND:**

Dengue, the disease caused by dengue virus (DENV), is responsible for over 300 million cases worldwide. Although DENV-4 usually presents with milder symptoms, some cases can result in death.

**OBJECTIVES:**

The present study aims to characterise the immunohistopathological profile of hepatic, pulmonary, cardiac and renal involvement in fatal cases of DENV-4 that occurred between 2011 and 2013 in Brazil.

**METHODS:**

To this end, tissue samples from the patients were subjected to histopathological analysis using bright-field microscopy and cytokine detection by immunohistochemistry.

**FINDINGS:**

Cells stained for tumour necrosis factor-alpha (TNF-α) and interferon-gamma (IFN-γ) were observed in the liver, lungs and kidneys. In the liver, the most common finding was periportal necrosis, along with steatosis and mononuclear cell infiltration. In the lungs, pneumocyte necrosis was only observed in areas of alveolar oedema. Septal and alveolar haemorrhage were present, along with thickening of the alveolar walls. In the kidneys, glomerular capillary congestion was observed in most of the analysed cases. Inflammatory cell infiltration was observed in the cortical region, along with proximal and distal tubule necrosis. Finally, in the heart, despite limited local cytokine expression, the cardiac tissue remained morphologically preserved.

**MAIN CONCLUSIONS:**

Although the findings reported in this study are consistent with those described in fatal dengue cases, the absence of cardiac alterations indicates that DENV-4 may cause a milder infection in this organ.

The dengue virus (DENV) belongs to the genus *Orthoflavivirus*, family *Flaviviridae*, which has four antigenically distinct serotypes: DENV-1, -2, -3 and -4.^1^ DENV is an arthropod-borne virus (arbovirus) transmitted by haematophagous mosquitoes of the *Aedes* genus, more specifically *Aedes aegypti* and *Aedes albopictus*. Infection with any of the four serotypes results in the disease known as dengue, an acute febrile illness characterised by its broad spectrum of clinical manifestations.^2^

DENV infection ranges from asymptomatic and subclinical cases to mild, self-limiting fever and very severe cases of haemorrhage and plasma leakage.^3^ When present, clinical manifestations occur after an incubation period of three to fifteen days.^4^ Although the vast majority of symptomatic cases present with mild symptoms such as fever, headache, retro-orbital pain, myalgia, arthralgia, leucopoenia and thrombocytopenia, around 5% of dengue infected patients develop the severe form of the disease, termed severe dengue (SD), a condition associated with increased vascular permeability, severe thrombocytopenia and haemorrhage in the skin and other organs.^5^ The evolution of SD can also lead to the development of hypotension, hypovolaemic shock and circulatory failure, which, if not treated promptly, can result in the patient's death within 8 to 24 h.^6^ Previous studies suggest an increased incidence of DENV infections resulting in the involvement of different systems, such as gastrointestinal, hepatic, respiratory, cardiac, neurological and renal, in adults and children.^7^ ^,^ ^8^ ^,^ ^9^ ^,^ ^10^

Dengue is prevalent in tropical and subtropical regions and is endemic in more than 120 countries. Epidemiological studies suggest that around 50 to 200 million people are infected annually, with 500,000 severe cases and more than 40,000 deaths associated with the disease.^2^ ^,^ ^11^ In Latin America alone, dengue is responsible for around 1.5 million cases every year,^12^ although it is estimated that almost half of the world's population lives in places where there is a real risk of infection.^13^ In Brazil, between the years of 1951-1960 and 2013-2022, the average transmission potential for dengue by *Ae. aegypti* has increased by 94.5%.^14^ This increase is the result of the interaction between various factors, such as climate change, population growth, uncontrolled urbanisation, migration, the evolution of the virus, inefficient health systems and socioeconomic inequality, among others.^15^ ^,^ ^16^

In 2023, 1,530,940 probable cases of dengue were detected in Brazil, which represents an increase of 16.5% in the number of cases compared to 2022. By epidemiological week 35 of 2023, 1,458 cases of SD had been confirmed, with 946 deaths from dengue.^17^ The number of probable cases further increased in 2024, with 6,215,201 detected between epidemiological weeks 1 and 26. During this same time period, 82,908 cases of SD were confirmed, an increase of 307.8% when compared to the previous year. The number of fatal cases of dengue also saw an increase in 2024, with 4,269 confirmed deaths.^18^ Regarding serotype distribution, DENV-1 represents the largest number of cases, with 39,816, followed by DENV-2, with 4,144, and lastly by DENV-3, with 46.^17^ ^,^ ^18^ Although DENV-4 has not been identified among the cases tested by the Brazilian Ministry of Health since 2018, circulation of this serotype can still be observed in the emergence of sporadic cases, such as in the states of Rio de Janeiro, Mato Grosso do Sul and Minas Gerais.^19^ ^,^ ^20^ ^,^ ^21^

In Brazil, although DENV-4 was first detected in 1981-82,^22^ in the northern region of the country, this serotype remained absent from the Brazilian epidemiological scenario for almost three decades, until it re-emerged in the State of Roraima, in 2010.^23^ After its reintroduction, DENV-4 quickly spread to other states in the country, favouring not only the increase of dengue cases in subsequent years, due to the high number of individuals susceptible to this new serotype, but also the risk of developing more severe manifestations in individuals previously infected with DENV-1, -2 or -3.^24^ While serotype 4 was not very prevalent among the cases analysed by the Ministry of Health of Brazil until 2015, its presence could be detected in more than two thirds of Brazilian states, and it has been responsible for a large number of epidemics since its reintroduction, mainly due to the population's susceptibility to the new serotype.^25^

DENV-4 cases usually present milder symptoms and are less associated with hospitalisations when compared to other serotypes.^26^ ^,^ ^27^ However, factors such as secondary infections by heterologous DENV serotypes^28^ or the interaction between dengue and other comorbidities often result in fatal cases,^29^ and can prove to be important aggravating factors during the course of the disease. In Brazil, the circulation of DENV-4 has been associated with mild cases,^30^ however, severe and fatal cases resulting from infection with this serotype have also been reported.^8^ ^,^ ^9^ ^,^ ^31^ In this context, this study aims to investigate the histopathological and inflammatory profile of four fatal cases of DENV-4, the latest serotype to be reintroduced in Brazil.

## MATERIALS AND METHODS

*Ethics statement* - All the procedures carried out in this study comply with the principles and regulations established by the resolution 196/96 of the National Health Council (Brazil) and were previously approved by the Human Research Ethics Committee of the Oswaldo Cruz Institute (Fiocruz) with Certificate of Submission for Ethical Appraisal Nº 57221416.0.1001.5248. The study was conducted in accordance with the Declaration of Helsinki, and approved by the Ethics Committee of Federal University of Ceará (Certificate of Submission for Ethical Appraisal Nº 58571016.0.0000.5054, approved 15/07/2017).

Liver, lung, heart and kidney samples from human fatal cases of dengue were kindly provided by Dr Fernanda Capelo Barroso Dr Emília Tomé de Souza from the Federal University of Ceará. These samples come from a project approved by the Human Research Ethics Committee of the Federal University of Ceará with Certificate of Submission for Ethical Appraisal Nº 58571016.0.0000.5054. Informed consent was obtained from all subjects and/or their legal guardian(s).

*Human fatal cases* - The liver, lung, heart and kidney samples analysed in this study were obtained from four fatal cases (Table) of dengue from the 2012 epidemic in the State of Ceará, and were received by spontaneous demand. Laboratory diagnosis with confirmation of the DENV-4 as the infecting serotype was previously carried out by the Central Laboratory of the State of Ceará through viral isolation (fresh tissue) followed by indirect immunofluorescence with specific monoclonal antibody for each serotype.^32^ Out of the four cases, only one, case #4, had been previously infected with DENV.

**TABLE:** Presentation of the four analysed fatal cases infected with dengue 4 (DENV-4)

	Age	Diagnostic confirmation	Comorbidities
Case #1	7	Immunohistochemistry	No
Case #2	47	Immunohistochemistry	No
Case #3*	70	Immunohistochemistry	Hypertension
Case #4	71	Immunohistochemistry and viral isolation	No

* Clinical diagnosis of severe dengue.

*Bright field microscopy* - The tissue samples were dehydrated in increasing concentrations of ethanol, clarified in xylene and embedded in paraffin. The resulting paraffin blocks were then sectioned into 4 µm slices using a microtome, mounted in glass slides, stained with haematoxylin and eosin (H&E) and finally analysed in a bright field microscope.

*Immunohistochemistry* - Tissue samples 4 µm thick were obtained in a microtome and mounted in glass slides. The peroxidase immunohistochemistry protocol was carried out as described by Rabelo et al.^33^ Briefly, these paraffin-embedded samples were deparaffinised and rehydrated. Antigen retrieval was performed by heating the tissue in citrate buffer (pH 6.0) at 90ºC. Subsequently, endogenous peroxidase activity was blocked and tissue sections were incubated with a Protein Blocker solution (Dako, USA). The samples were then incubated overnight with the primary antibodies tumour necrosis factor-alpha (TNF-α) (Sc-52746; Santa Cruz Biotechnology, TX, USA) or interferon gamma (IFN-γ) (250707; Abbiotec, CA, USA) at a concentration of 1:200 in a dark chamber at 4ºC. The following day, the tissue samples were incubated with a secondary horseradish peroxidase-conjugated antibody (Spring Bioscience, USA) for 15 min. The reaction was revealed using diaminobensidine as a chromogen and the sections were counterstained with Harris' haematoxylin. For negative control of the immunohistochemistry reaction, samples were incubated only with the secondary horseradish peroxidase-conjugated antibody.

## RESULTS

**Fig. 1:**
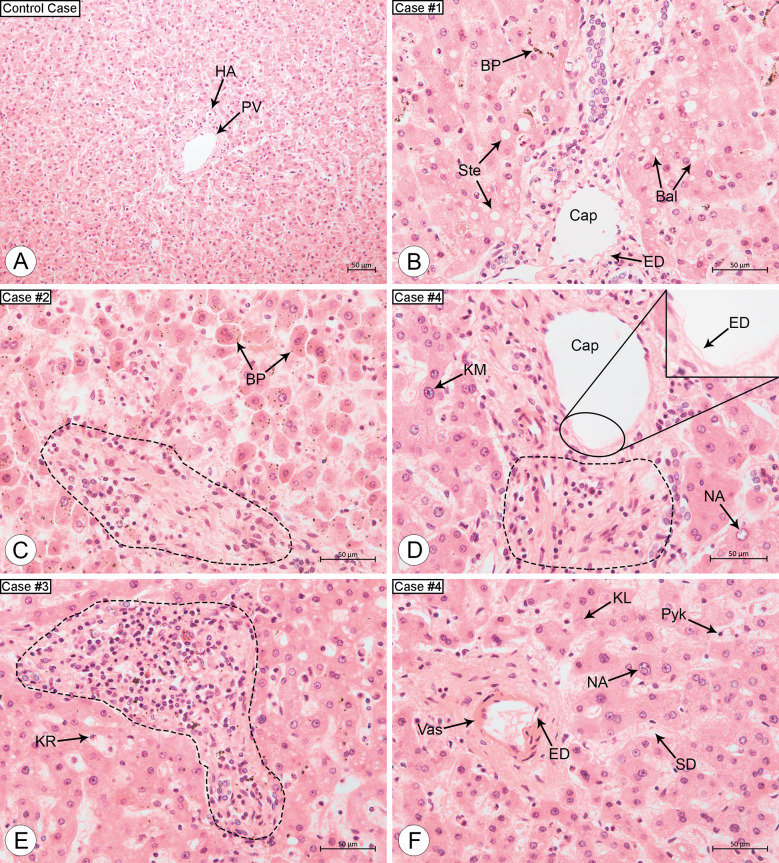
micrographs of liver sections stained with haematoxylin and eosin. (A) Liver of a patient not infected with dengue virus 4 (DENV-4). HA: hepatic artery; PV: portal vein (B, C, D, E, F). Livers of patients infected with DENV-4. Bal: ballooning degeneration; BP: biliary pigment; Cap: capillary; dashed lines: areas of hepatocyte necrosis; ED: endothelial detachment; HA: hepatic artery; KL: karyolysis; KM: karyomegalia; KR: karyorrhexis; NA: nuclear atypia; Pyk: pyknosis; SD: sinusoidal capillary dilation; Ste: steatosis; Vas: vasculitis. Magnification: A: 200X; B, C, D, E, F: 400X.

**Fig. 2:**
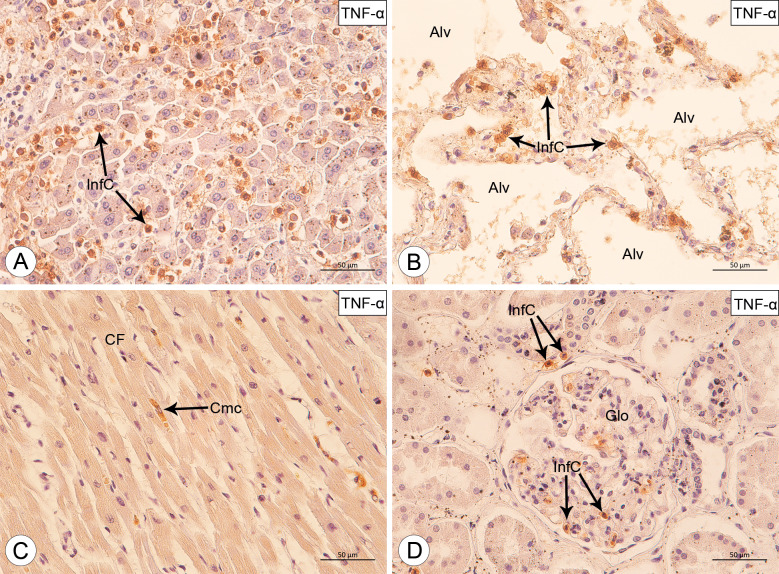
micrographs of immunostained organs incubated with anti-tumour necrosis factor-alpha (TNF-α) antibody. (A) Liver of a patient infected with dengue virus 4 (DENV-4). InfC: inflammatory cell stained for TNF-α. (B) Lung of a patient infected with DENV-4. Alv: alveolus. (C) Heart of a patient infected with DENV-4. CF: cardiac fibres; Cmc: cardiomyocyte stained for TNF-α. (D) Kidney of a patient infected with DENV-4. Glo: glomerulus. Magnification: A, B, C, D: 400X.

**Fig. 3:**
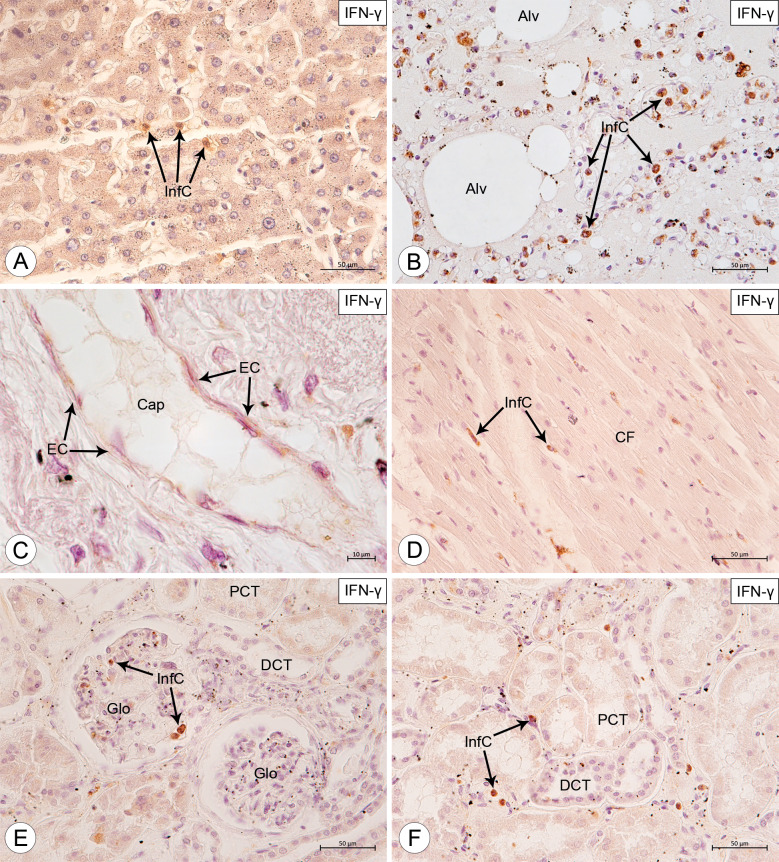
micrographs of immunostained organs incubated with anti-interferon-gamma (IFN-γ) antibody. (A) Liver of a patient infected with dengue virus 4 (DENV-4). InfC: inflammatory cell stained for IFN-γ. (B, C) Lungs of patients infected with DENV-4. Alv: alveolus; Cap: capillary; EC: endothelial cell. (D) Heart of a patient infected with DENV-4. CF: cardiac fibres. (E, F) Kidneys of patients infected with DENV-4. Glo: glomerulus; DCT: distal convoluted tubule; PCT: proximal convoluted tubule. Magnification: C: 400X; A, B, D, E, F: 200X.

*Histopathology of the liver of DENV-4 infected fatal cases* - The main finding in the liver was necrosis (Fig. 1C-E). These extensive areas of hepatocytes undergoing the process of cell death were predominantly located in the centrilobular zone of the hepatic lobule. Also in this zone, signs of ballooning degeneration could be observed in hepatocytes (Fig. 1B), as well as dilation of the sinusoidal capillaries (Fig. 1F). Round, well-defined vacuoles were present in the cytoplasm of hepatocytes (Fig. 1B). This finding, called steatosis, signals the excessive accumulation of lipids inside the cell, usually causing lateralisation of the nucleus, and was often present in regions of hepatocyte necrosis. Hepatocytes near areas of necrosis had biliary pigments accumulated in the cytoplasm (Fig. 1B-C), which, in higher concentrations, can lead to cell death. Different types of nuclear degeneration, such as karyorrhexis (Fig. 1E), karyolysis (Fig. 1F) and pyknosis (Fig. 1F) were observed in necrotic hepatocytes. Many nuclei presented irregular morphology (Fig. 1F), and karyomegaly was observed in many hepatocytes (Fig. 1D). Vasculitis was observed in some capillaries (Fig. 1F), often with the detachment of the endothelial wall (Fig. 1B, D). Mononuclear cell infiltration was also observed in areas of necrosis, with the presence of Kupffer cells (Fig. 1C-E). Furthermore, the cytokine TNF-α was detected in a considerable proportion of inflammatory cells in the sinusoidal capillaries (Fig. 2A), while IFN-γ was also detected, albeit in fewer inflammatory cells (Fig. 3A). No areas of haemorrhage were observed in any of the analysed livers. Overall, hepatic morphology of the studied areas was significantly altered, particularly due to the disruption of the parenchymal integrity, caused by the necrosis of the tissue.

In contrast, the liver of a patient that was not infected with DENV-4 presented well-defined hepatic lobules, with uniform hepatic cords and healthy hepatocytes. The structures of the triad region had a normal aspect, with no alterations in the morphology of the biliary duct, the hepatic artery or the portal vein (Fig. 1A). The central vein and the surrounding parenchyma also presented the regular aspect of a well-functioning liver.

**Fig. 4:**
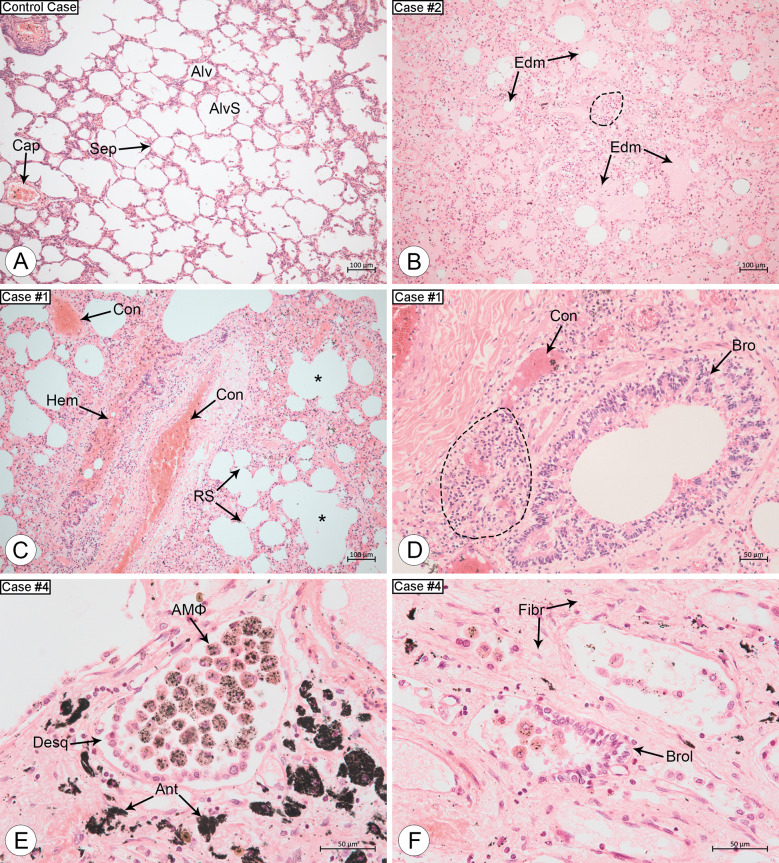
micrographs of lung sections stained with haematoxylin and eosin. (A) Lung of a patient not infected with dengue virus 4 (DENV-4). Cap: capillary; Sep: septum; Alv: alveolus; AlvS: alveolar sac. (B, C, D, E, F) Lungs of patients infected with DENV-4. Asterisk (*): compensatory alveolar hyperinflation; AMΦ: alveolar macrophage; Ant: anthracosis; Bro: bronchitis; Brol: bronchiolitis; Con: vascular congestion; Dashed line: inflammatory cell infiltration; Desq: cell desquamation; Edm: oedema; Fib: fibrosis; Hem: haemorrhage; RAS: ruptured septum. Magnification: A, B, C: 100X; D: 200X; E, F: 400X.

*Histopathology of the lung of DENV-4 infected fatal cases* - In the lung, several areas of oedema were observed, sometimes completely occluding the alveolar space (Fig. 4B). This exudate presented small lymphocytic content, with alveolar macrophages in its midst. In some analysed fields, few alveoli were unobstructed, leaving a diminished functional area for gas exchange. In addition, blood vessels of large and small calibre showed signs of vascular congestion, with alveolar macrophages among the stagnant erythrocytes (Fig. 4C-D). The cytokine IFN-γ was detected in endothelial cells close to areas of inflammatory cell infiltration (Fig. 3C). Thickening of the alveolar septa was widely observed, mainly due to the infiltration of mononuclear cells (Fig. 4B, D) and to haemorrhage (Fig. 4C), as well as ruptured alveolar septa (Fig. 4C), bronchitis (Fig. 4D) and bronchiolitis (Fig. 4F), which would further impair the gas exchange. Due to this, many alveoli showed signs of compensatory hyperinflation, being overly expanded (Fig. 4C). The presence of TNF-α and IFN-γ in inflammatory cells in the pulmonary parenchyma was confirmed through immunostaining (Figs 2B, 3B). The presence of alveolar macrophages was observed in the alveolar septa, the alveolar space and in the respiratory bronchioles (Fig. 4E). The desquamation of the bronchiolar epithelium was a common finding (Fig. 4E). In one particular case (case #4), carbon particles were observed, both deposited in the bronchiolar interstitium and phagocytosed by alveolar macrophages, with the appearance of anthracosis (Fig. 4E), possibly associated with the patient's lifestyle. Extensive areas of fibrosis were also present in this case (Fig. 4F).

The lung of a patient that was not infected with DENV-4 presented healthy bronchioles and alveolar sacs, with unobstructed alveoli and thin septa (Fig. 4A). No noteworthy morphological alterations were observed in the pulmonary parenchyma. Capillaries and blood vessels also had a normal aspect.

**Fig. 5:**
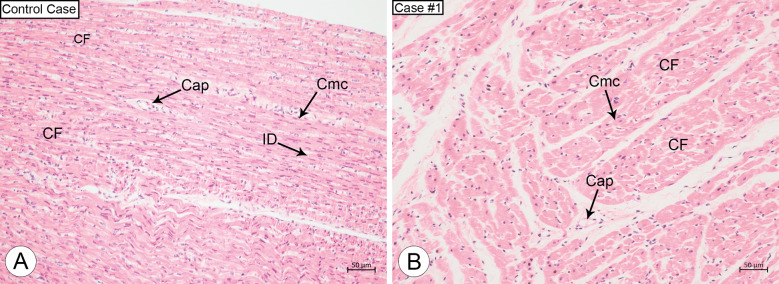
micrographs of heart sections stained with haematoxylin and eosin. (A) Heart of a patient not infected with dengue virus 4 (DENV-4). Cap: capillary; CF: cardiac fibres; Cmc: cardiomyocyte; ID: intercalated disk. (B) Heart of a patient infected with DENV-4. Magnification: A, B: 200X.

*Histopathology of the heart of DENV-4 infected fatal cases* - Overall, the analysed hearts presented normal morphology, with no alterations indicating a pathological process. The cardiac fibres, the cardiomyocytes and the capillaries, showed no abnormal characteristics (Fig. 5B). Very few cardiomyocytes in the cardiac parenchyma were stained for TNF-α and, in a similar scenario, few inflammatory cells stained for IFN-γ were observed (Figs 2C, 3D).

Likewise, the heart of a patient that was not infected with DENV-4 presented a healthy appearance, with a similar morphology to the infected patient (Fig. 5A).

**Fig. 6:**
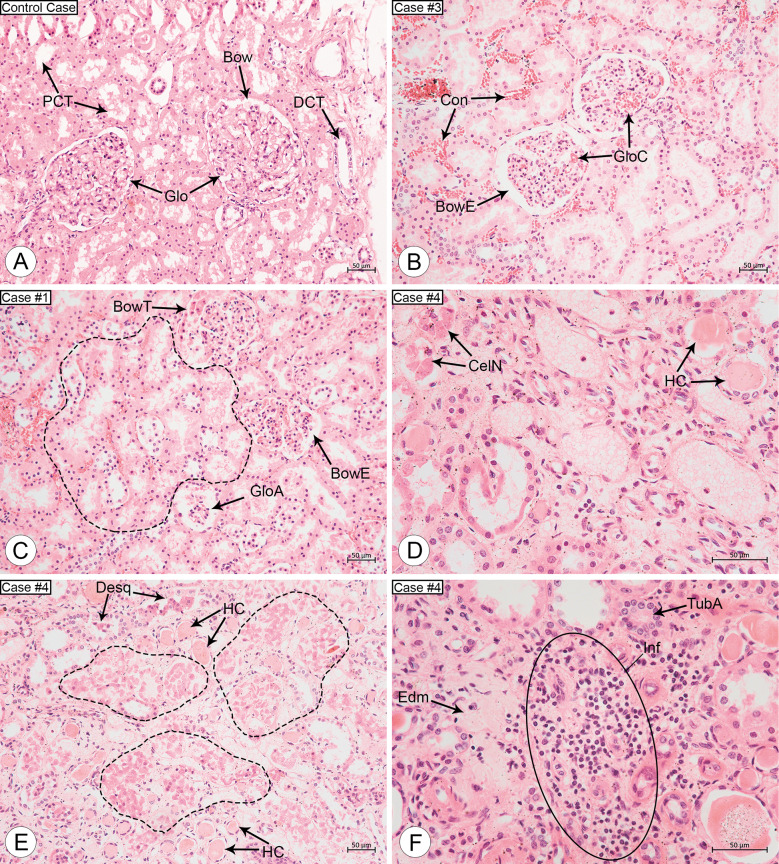
micrographs of kidney sections stained with haematoxylin and eosin. (A) Kidney of a patient not infected with dengue virus 4 (DENV-4). Bow: bowman's space; DCT: distal convoluted tubule; Glo: glomerulus; PCT: proximal convoluted tubule. (B, C, D, E, F) Kidneys of patients infected with DENV-4. BowE: bowman's space enlargement; BowT: bowman's capsule thickening; CelN: tubular cell necrosis; Dashed line: area of tubular necrosis; Desq: cell desquamation; Edm: oedema; GloA: glomerular atrophy; GloC: glomerular congestion; HC: hyaline cast; Inf: inflammatory cell infiltration; TubA: tubular atrophy. Magnification: A, B, C, E: 200X; D, F: 400X.

*Histopathology of the kidney of DENV-4 infected fatal cases* - In the cortical region of the kidney, the structure of the Malpighian corpuscle was noticeably altered, with signs of glomerular capillary congestion (Fig. 6B), glomerular atrophy (Fig. 6C), enlargement of the Bowman's space (Fig. 6B-C) and thickening of the parietal epithelium of the Bowman's capsule (Fig. 6C). The most common finding was tubular necrosis, with the death of tubular cells that form the epithelium of both the proximal and distal convoluted tubules (Fig. 6C-E). These areas of necrosis would often be close to morphologically altered Malpighian corpuscles. The degree of tubular damage varied, with some tubules only presenting a partial desquamation of the epithelium (Fig. 6E), while others were devoid of functional cells and entirely filled with cellular debris (Fig. 6C). Tubular atrophy was also observed, with extreme reduction of the luminal space (Fig. 6F). The formation of hyaline casts, structures composed of condensed plasma proteins, was regularly observed in the lumen of renal tubules (Fig. 6D-6E), suggesting an altered capillary permeability in the glomerulus. Infiltration of inflammatory cells was observed in some cases, close to areas of tubular necrosis and oedema (Fig. 6F), but was sparse and not present throughout the entirety of the renal parenchyma. Still, some inflammatory cells stained for TNF-α and IFN-γ were detected both in the glomerular capillaries and in renal capillaries (Figs 2D, 3E-F). The medullary region of the kidney presented a regular morphology, with no noteworthy alterations.

In comparison, the kidney of a patient that was not infected with DENV-4 showed a regular morphology, made up of healthy Malpighian corpuscles and a normal tubular structure, with no pathological alterations present (Fig. 6A).

## DISCUSSION

During DENV infection, alterations in the liver are among the most commonly observed and are usually associated with cases of SD.^34^ ^,^ ^35^ Human patients present jaundice, acute hepatitis, hepatomegaly, liver failure and elevated serum albumin levels. Macroscopically, several areas of vascular congestion as well as several haemorrhagic foci can be observed. In bright field microscopy, several areas of inflammatory infiltrate can be seen near the portal space, as well as focal vacuolisation of the cytoplasm of hepatocytes, areas of vascular congestion, the presence of inflammatory infiltrate in sinusoidal capillaries, hyperplasia of Kupffer cells and lipid inclusions in hepatocytes.^7^ ^,^ ^36^ ^,^ ^37^ Other alterations normally observed on the cellular level include micro and macrogoticular steatosis, focal areas of necrosis with presence of a mononuclear inflammatory infiltrate, mitochondrial engorgement, characteristic of the apoptotic process, haemorrhage and oedema.^7^

In this study, histopathological analysis of the livers of fatal human cases revealed classic findings of dengue, such as steatosis, inflammatory cell infiltration, hepatocyte necrosis and dilation of sinusoidal capillaries. Kupffer cell hyperplasia, which has been previously described in human fatal cases of dengue,^7^ is certainly a result of the increased activity of these cells during DENV infection, since macrophages play a large role in activating the inflammatory response and vasodilation through the release of cytokines and chemokines.^38^ Dilation of the sinusoidal capillaries, generally located in the periportal or perivascular region, suggests a predisposition related to vascularisation disorders.^39^ It is known that hypertension in the central vein can result in an increase in sinusoidal fenestrae, a reduction in portal venous inflow and, as a consequence of the change in pressure change, promote periportal necroinflammatory activity.^40^ Furthermore, the obstruction of the portal vein, leading to a decreased blood flow velocity, has also been associated with the dilation of the sinusoidal capillaries.^41^ The accumulation of biliary pigments in the cytoplasm of hepatocytes, while not a widespread alteration, could indicate an initial presentation of cholestasis, which has been described as a rare hepatic manifestation caused by dengue infection.^42^ Balloon-like degeneration of hepatocytes is one of the main findings in non-alcoholic steatohepatitis,^43^ although it is not a commonly described finding in dengue. This form of cell death may be associated with a late and irreversible stage of lipid accumulation in the cytoplasm.^44^

Pulmonary manifestations in human cases of DENV infection are uncommon, however, alterations such as peribronchial thickening, bilateral pulmonary congestion, pleural effusion, pulmonary oedema, haemorrhage and pneumonitis have already been reported in cases of dengue and SD.^45^ ^,^ ^46^ ^,^ ^47^ Atypical manifestations include acute respiratory distress syndrome and pulmonary dysfunction.^37^ Histopathological and ultrastructural analysis of lung tissue from fatal cases of dengue revealed interstitial pneumonia, thickening of the alveolar septum, diffuse infiltration of mononucleated and polymorphonucleated cells, areas of alveolar congestion, appearance of a hyaline membrane, type II pneumocyte hyperplasia, platelet recruitment, areas of haemorrhage and oedema, and the presence of viral particles in the endothelium.^7^ ^,^ ^9^ ^,^ ^36^ ^,^ ^45^

The most consistent findings in the fatal cases analysed in this study were observed in the lungs. Haemorrhage, septal thickening, characterising pneumonitis, inflammatory cell infiltration and vascular congestion were observed in all the cases of death. Curiously, the presence of oedema was not detected in the case diagnosed with SD. Even so, in this specific case, vascular congestion was observed in almost all the vessels analysed, suggesting the presence of alterations in the pulmonary circulatory system. Pulmonary fibrosis, observed in one of the fatal cases, is a chronic regenerative process associated with a range of factors, such as smoking, gastro-oesophageal reflux, the use of some medications, diabetes mellitus, exposure to occupational risks and various infectious agents, such as the Epstein-Barr virus, cytomegalovirus and human herpes viruses 7 and 8,^48^ although it has never been directly associated with DENV infection. Pulmonary haemorrhage was also observed in some of the cases, most notably in the interalveolar septum, though it was also present in the alveoli, which could have led to the ruptured septa observed. Pulmonary haemorrhage has already been reported in fatal human cases,^7^ ^,^ ^9^ ^,^ ^36^ usually associated with haemoptysis.^45^ ^,^ ^49^ It is important to note that the presence of oedema is not necessarily a histopathological finding associated with DENV infection, and may be a common complication of fluid replacement in dengue patients.^50^ Even so, oedema, accompanied by haemorrhage, is a finding suggestive of alterations in vascular permeability and of plasma.^51^

To date, the symptomatology and histopathology of the manifestations caused in the heart by DENV are still poorly studied.^52^ Abnormal aspects of cardiac function observed in DENV infection include sinus tachycardia, increased jugular pressure, altered heart rhythm and interstitial oedema,^53^ as well as bilateral ventricular systolic dysfunction, the latter observed more commonly in cases of SD.^54^ Cases of myocarditis are rare in DENV infections and are often asymptomatic, although they are among the most common findings observed in cardiac tissue.^55^ ^,^ ^56^ Myocarditis can have serious clinical consequences, such as arrhythmia, heart failure and cardiogenic shock.^57^ ^,^ ^58^ The X-ray of a human patient with a confirmed case of dengue revealed progressive cardiomegaly^59^ ^,^ ^60^ and histopathological and ultrastructural analysis of cardiac tissue from fatal cases of dengue revealed the presence of nuclear and mitochondrial alterations, degeneration of cardiac fibres, haemorrhage, interstitial oedema, diffuse inflammatory infiltrate and the presence of macrophages in the myocardium.^7^ ^,^ ^52^

In the fatal cases analysed in this study, no noteworthy alterations were observed in the heart. This may be explained by the rarity of cardiac involvement during dengue,^37^ ^,^ ^61^ since most patients do not show myocardial damage or signs of cardiomyocyte infection by the virus.^54^

Even though the role of the kidneys during dengue infection is not thoroughly understood, serious manifestations such as acute kidney injury and glomerulonephritis have been reported.^62^ ^,^ ^63^ It is believed that renal damage can be caused by multiple factors, such as haemolysis, haemodynamic alterations induced by the virus, deposition of immune complexes in the glomerulus and deposition of muscle proteins resulting from rhabdomyolysis.^63^ ^,^ ^64^ ^,^ ^65^ Histopathological alterations include congestion of glomerular capillaries, focal haemorrhage, oedema, inflammatory cell infiltration, hydropic degeneration, micro abscess formation, thrombus formation in the glomerulus, and acute tubular necrosis.^7^ ^,^ ^8^ ^,^ ^9^ ^,^ ^66^ Acute kidney injury, a condition characterised by a sudden drop in kidney function, is one of the most frequently reported findings, although the factors that cause its onset during dengue are mostly unexplored.^67^ ^,^ ^68^ ^,^ ^69^ The histopathology of the kidney during dengue is characterised by necrosis of the proximal and distal tubules, with dilation of the lumen, loss of the brush border, simplification of the tubular epithelium and loss of the nucleus.^70^

In this study, tubular necrosis was observed in all cases analysed. Even though the viral antigen has been previously detected in the epithelium of tubular cells,^71^ the capacity of DENV to infect these cells has not been demonstrated yet. It has been suggested that the damage to the renal tubules is caused by ischaemia, due to a decreased blood flow to the kidney, on account of the significant blood volume loss during SD.^7^ In a likely manner, tubular atrophy, characterised by a notable reduction of the tubular lumen, is also associated with the ischaemic process of small blood vessels of the kidney.^72^ Also observed was the formation of hyaline casts in the tubular space, which is a common occurrence in the kidney. The ones observed in this study appear to be formed mostly by uromodulin — a strong immunomodulatory protein — due to their well-defined round morphology and pale-pink colour when stained with H&E. While normally described as a benign process, hyaline cast formation has been previously associated with tubular and glomerular injury.^72^ ^,^ ^73^ ^,^ ^74^ In turn, the obstruction of the renal tubules, be it by the reduction of the lumen due to atrophy or the overaccumulation of proteins, may alter the hydrostatic pressure gradient across the glomerular capillaries, leading to an enlargement of the Bowman's space.^75^ The congestion of the glomerular capillaries is possibly the result of the endothelial alterations caused by SD and are probably aggravated by the atrophy of the glomerulus.^63^ ^,^ ^76^ It is also possible that the reduction of the glomerular lumen facilitates the deposition of immune complexes in the glomerulus. Although the importance of immune complexes during dengue-related kidney disease has been widely discussed,^76^ ^,^ ^77^ ^,^ ^78^ ^,^ ^79^ a clear consensus has yet to be established on how exactly the pathological process occurs.

Finally, also in this study, a large number of mononuclear inflammatory cells marked for TNF-α were detected in liver, lung and kidney of DENV-4 infected patients. Inflammatory cells marked for IFN-γ were also detected in these same organs, albeit to a lesser degree. In the lung, specifically, endothelial cells were marked for IFN-γ.

Various cytokines and chemokines are related to tissue damage, being secreted by macrophages and T lymphocytes during the attempt to contain DENV infection.^80^ ^,^ ^81^ Furthermore, TNF-α is capable of altering the vascular permeability of capillaries, triggering cases of plasma leakage,^82^ and is detected in higher levels during secondary DENV infections. Likewise, IFN-γ levels are also higher during secondary DENV infection and during SD.^83^ ^,^ ^84^ ^,^ ^85^ While it has been suggested that IFN-γ secretion plays an important part in the hyperpermeability so characteristic of SD,^86^ this cytokine appears to be downregulated by DENV replication and viral RNA production.^87^ Additionally, a previous study conducted in mice lacking interferon receptors demonstrated that IFN-γ plays a role in eliciting a protective response from the initial systemic dissemination of DENV following infection.^88^ Even still, these cytokines seem to correlate with disease severity and could be used as disease severity markers.^89^

In the analysed fatal human cases infected with DENV-4, the findings were consistent with what is described in the literature, particularly regarding hepatic, pulmonary and renal alterations. Still, no cardiac alterations were observed, reinforcing either their rarity or a diminished tropism of this serotype for heart cells.

Although hepatic histopathological manifestations are among the most commonly reported during DENV-4 infection, in this study isolated areas of hepatocyte necrosis and inflammation were observed, suggesting a milder infection in the liver. Furthermore, due to severe alterations in the lung, such as haemorrhage and oedema, which can prove fatal even when properly treated, this study suggests that pulmonary manifestations may also play an important role in more severe cases of dengue.

The kidney, on the other hand, is, supposedly, not a viral replication site, but is still largely affected by the systemic manifestations of dengue, presenting tubular and glomerular alterations that can lead to renal failure. Furthermore, even though DENV-4 infection is said to cause a milder form of the disease, this study shows that it is still able to evolve into more severe cases of dengue, that can even lead to death.

Despite being absent from the epidemiological reports of the past four years in Brazil, isolated sporadic cases of DENV-4 have been identified throughout the country, indicating a silent circulation, that goes mostly undetected due to the high prevalence of other serotypes. Ultimately, despite being less associated with hospitalisations and deaths when compared to other serotypes, DENV-4 should still be treated as one of the four cornerstones of dengue, equally as important as DENV-1, DENV-2 and DENV-3, particularly due to factors such as secondary infections with heterologous serotypes or the other comorbidities that can often result in fatal cases.

The main limitation of this study is the limited clinical information and lack of in-depth laboratory diagnosis of the DENV-4 fatal cases. Unfortunately, since the samples were received by spontaneous demand, long after the end of the epidemic, it is not possible for this data to be retrieved.

## Data Availability

The data that supports the findings of this study were generated at Oswaldo Cruz Foundation/Oswaldo Cruz Institute and is available from the corresponding author on request.
