# Supplementary material for: Dengue virus serotype 4 infection in human fatal cases: histopathological investigation
Source: Mem Inst Oswaldo Cruz. 2026 Jun 22;121:e250248. doi: 10.1590/0074-02760250248 (PMC13313159; doi:10.1590/0074-02760250248)
Supplement: Peer review history

# Reviewer report: FIRST REVIEW ROUND - REVIEWERS COMMENTS

## REVIEWER #1

This is an interesting manuscript aiming to investigate the histopathological and inflammatory profile of four fatal cases of DENV-4.

I have some questions related to methods and cases.

- At first, I understood that all four patients had DENV-4.

However, in lines 113,114, one of them had other sorotypes. So there are 3 with DENV-4 and 1 without DENV-4?

- Why do the authors chose only TNF-alpha and IFN-gamma to demonstrate inflammation?

-Lines 318 and 319, correct: IFN (not INF)

Line 68 - check the number (6.215.20?)

Line 93 - put in order: 8,9,31

Line 122 - What was th temperature of antigen retrieval?

Line 152 - Please add the code and brand of antibodies

## REVIEWER #2

Rasinhas et al. describe histopathological findings in multiple organs from four DENV-4–positive patients.

While the study addresses an important topic—given that little is known about dengue pathogenesis and no animal model fully reproduces human disease—the manuscript lacks some key information that would strengthen its impact and clarity.

Additional details on methodology, as well as minor improvements in organization and terminology, are needed to enhance the overall quality and scientific rigor of the work.

The abstract is generally well structured and clearly summarizes the study objectives, methods, and main findings.

The topic is relevant and contributes to understanding the organ-specific pathology associated with DENV-4 infection.

However, I don’t think the last sentence regarding findings in the heart is necessary.

I would change to “while cardiac tissue remained morphologically preserved despite limited local cytokine expression.”

The introduction is well structured and provides sufficient background on dengue pathogenesis.

However, in line 37, the authors should update the Orthoflavivirus nomenclature according to the latest ICTV classification.

The methodology section requires more detailed description. For example, the authors do not specify how DENV-4 was confirmed after cell isolation—was it by immunohistochemistry, PCR, immunofluorescence assay, sequencing, or another method?

Additionally, they mention that one patient had a secondary dengue infection; it is unclear how this was determined.

Please provide details on the diagnostic criteria or assays used to identify secondary infection.

Clarifying these points will improve the reproducibility and rigor of the study.

The results section provides valuable insights into the histopathological findings across multiple organs from four patients.

However, it is unclear whether the reported findings were observed in all patients or varied between individuals.

Were there differences related to patient age or the presence of a secondary dengue infection?

Clarifying this information would help to better understand the organ-specific lesions associated with secondary infections.

I also recommend including a summary table that compiles the main histopathological findings for each organ and each patient, which would enhance clarity and facilitate comparison.

The discussion section needs to be revised.

The statement “Although the liver is a primary target for DENV-4 infection” (lines 336-338) should be revised for accuracy.

Current evidence indicates that while the liver is an important target organ affected during DENV infection, it is not necessarily the primary site of viral replication.

Early viral replication typically occurs in mononuclear phagocytic and endothelial cells before systemic dissemination.

Also, the sentence “it appears that the lungs are more susceptible to the virus, presenting histopathologic alterations, namely hemorrhage and edema, that can prove fatal even when properly treated” should be reconsidered.

While pulmonary involvement (e.g., edema, hemorrhage, and alveolar damage) has been reported in severe dengue cases, the lungs are not generally considered more susceptible to DENV infection than other organs.

Additionally, some in-text references within parentheses appear out of order.

Please review and ensure that all citations are correctly numbered and sequential.

## AUTHORS’ RESPONSE TO THE REVIEWERS

Response to Reviewers

Reviewer: 1

- At first, I understood that all four patients had DENV-4.

However, in lines 113,114, one of them had other sorotypes. So there are 3 with DENV-4 and 1 without DENV-4?

**Author’s Reply:** To clarify, all four patients were infected with DENV-4, but only one patient had been previously infected by DENV (confirmed via ELISA, with anti-DENV IgG detection).

The three remaining patients had never been infected with DENV before. This section has been revised and corrected (lines 111-114).

- Why do the authors choose only TNF-alpha and IFN-gamma to demonstrate inflammation?

**Author’s Reply:** Unfortunately, other significant pro inflammatory cytokines known to have a role during DENV infection, such as IL-6 and IL-8 were not readily available, and for that reason we opted to use only TNF-α, which is a central mediator of inflammation and IFN-γ, which modulates antiviral response, to evaluate the inflammatory response.

-Lines 318 and 319, correct: IFN (not INF)

**Author’s Reply:** The section has been revised and corrected.

Line 68 - check the number (6.215.20?)

**Author’s Reply:** The section has been revised and corrected.

Line 93 - put in order: 8,9,31

**Author’s Reply:** The section has been revised and corrected.

Line 122 - What was th temperature of antigen retrieval?

**Author’s Reply:** The antigen retrieval was performed at 90ºC. This information has been added to the manuscript (line 122).

Line 152 - Please add the code and brand of antibodies

**Author’s Reply:** The code and brand of each antibody is as follows - TNF-α: Sc-52746 (Santa Cruz Biotechnology, TX, USA);

IFN-γ: 250707 (Abbiotec, CA, USA). This information has been added to the manuscript (lines 124-126).

## Reviewer: 2

- The abstract is generally well structured and clearly summarizes the study objectives, methods, and main findings.

The topic is relevant and contributes to understanding the organ-specific pathology associated with DENV-4 infection.

However, I don’t think the last sentence regarding findings in the heart is necessary.

I would change to “while cardiac tissue remained morphologically preserved despite limited local cytokine expression.”

**Author’s Reply:** The section has been revised and rewritten (lines 31-32).

- The introduction is well structured and provides sufficient background on dengue pathogenesis.

However, in line 37, the authors should update the Orthoflavivirus nomenclature according to the latest ICTV classification.

**Author’s Reply:** According to ICTV, dengue virus (Orthoflavivirus denguei) is classified as belonging to the Flaviviridae family and the Orthoflavivirus genus.

This information is already included in the text (Line 37).

- The methodology section requires more detailed description. For example, the authors do not specify how DENV-4 was confirmed after cell isolation — was it by immunohistochemistry, PCR, immunofluorescence assay, sequencing, or another method?

**Author’s Reply:** Serotype confirmation following viral isolation was performed through indirect immunofluorescence, performed by the Central Laboratory of the State of Ceará.

This information has been added to the manuscript (Lines 111-114).

- Additionally, they mention that one patient had a secondary dengue infection; it is unclear how this was determined.

Please provide details on the diagnostic criteria or assays used to identify secondary infection.

Clarifying these points will improve the reproducibility and rigor of the study.

**Author’s Reply:** Detection of DENV antibodies was performed by the Central Laboratory of the State of Ceará, through ELISA.

Anti DENV-IgG detection confirmed the patient had previously been infected by a different serotype of DENV.

This section has been revised and corrected (lines 113-114).

- The results section provides valuable insights into the histopathological findings across multiple organs from four patients.

However, it is unclear whether the reported findings were observed in all patients or varied between individuals.

Were there differences related to patient age or the presence of a secondary dengue infection?

Clarifying this information would help to better understand the organ-specific lesions associated with secondary infections.

I also recommend including a summary table that compiles the main histopathological findings for each organ and each patient, which would enhance clarity and facilitate comparison.

**Author’s Reply:** Although the intensity of some findings, such as necrosis, steatosis, edema, hemorrhage and tubular necrosis varied across all cases, there were no significant differences in findings regarding either patient age or secondary infection.

Even though case #4 presented a secondary DENV infection, the apparent severity of the infection could not be perceived in the tissue, as the profile was similar among all cases.

Most alterations described were observed in all cases, and when not, this was specified in the text, such as the finding of an excessive amount of carbon particles in the lung of case #4.

This information has been added to the text (line 174).

Additional information regarding the histopathology micrographs, i.e. to which case each image belongs to (figures 1, 2, 3 and 4).

Still, due to the qualitative nature of the study we have opted to maintain a more descriptive approach.

- The discussion section needs to be revised. The statement “Although the liver is a primary target for DENV-4 infection” (lines 336-338) should be revised for accuracy.

Current evidence indicates that while the liver is an important target organ affected during DENV infection, it is not necessarily the primary site of viral replication.

Early viral replication typically occurs in mononuclear phagocytic and endothelial cells before systemic dissemination.

Also, the sentence “it appears that the lungs are more susceptible to the virus, presenting histopathologic alterations, namely hemorrhage and edema, that can prove fatal even when properly treated” should be reconsidered.

While pulmonary involvement (e.g., edema, hemorrhage, and alveolar damage) has been reported in severe dengue cases, the lungs are not generally considered more susceptible to DENV infection than other organs.

**Author’s Reply:** The paragraph has been rewritten (lines 337-342).

- Additionally, some in-text references within parentheses appear out of order.

Please review and ensure that all citations are correctly numbered and sequential.

**Author’s Reply:** This information has been revised and corrected.

# Reviewer report: REVIEWERS COMMENTS

## REVIEWER #1

Authors have addressed all reviewers questions and is now suitable for publication.

However, reference number 32 for the IFA test is actually Gubler, D. J., Kuno, G., Sather, G. E., Velez, M. & Oliver, A. Mosquito cell cultures and specific monoclonal antibodies in surveillance for dengue viruses.

Am. J. Trop. Med. Hyg. 33, 158–165 (1984). Please correct.

## REVIEWER #2

I believe all the considerations that were previously mentioned were adequately answered and corrected in the new version of the manuscript.
